# Metabolomic profiling of neat uterine luminal fluid in cows: local enrichment and nutritional modulation

**DOI:** 10.1080/01652176.2025.2611849

**Published:** 2026-01-07

**Authors:** Masroor Sagheer, Quinn A. Hoorn, Mariangela BC. Maldonado, Kasey M. Schalich, Nadia Ashrafi, Romana A. Mimi, Stewart F. Graham, Vimal Selvaraj, Peter J. Hansen

**Affiliations:** a Department of Animal Sciences, Institute of Food and Agricultural Sciences, D.H. Barron Reproductive and Perinatal Biology Research Program, and Genetics Institute, University of Florida, Gainesville, FL, USA; b Department of Animal Science, Cornell University, Ithaca, NY, USA; c Metabolomics Department, Beaumont Research Institute, Royal Oak, MI, USA; d Corewell Health East William Beaumont University Hospital, Royal Oak, MI, USA; e Oakland University-William Beaumont School of Medicine, Rochester, MI, USA

**Keywords:** Uterus, cow, metabolome, estrous cycle

## Abstract

Uterine luminal fluid influences embryonic development and the subsequent phenotype of offspring, yet its detailed metabolomic composition remains poorly characterized. Here, minimally invasive transcervical techniques were employed to collect neat uterine fluid from postpartum dairy cows and cyclic beef cows to allow for metabolomic profiling *via* targeted mass spectrometry. Objectives were to 1) compare the metabolomic profile of uterine fluid with plasma in dairy cows and 2) assess the impact of dietary rumen-protected methionine and stage of estrous cycle (day 0 vs 7) on plasma and uterine fluid metabolomic profile in beef cows. Results revealed that the concentrations of many metabolites, including amino acids, signaling molecules (e.g. dopamine, gamma-aminobutyric acid) and lipids (e.g. ceramides, diacylglycerols), were higher in uterine fluid than in plasma. An oral bolus of rumen-protected methionine increased uterine concentration of methionine on day 0 of the estrous cycle. The uterine metabolome remained relatively stable between days 0 and 7 although there was temporal variability for a select number of metabolites (cysteine, methionine, methionine sulfoxide, asymmetric dimethylarginine, ceramides, and glycerophospholipids). Correlations between plasma and uterine fluid concentrations were strong or moderate for many amino acids. Collectively, these findings highlight that the uterine lumen is a specialized, selectively regulated biochemical compartment.

## Introduction

The successful development of a preimplantation embryo is dependent on the uterine luminal milieu. This environment is shaped through the integration of systemic factors derived from the maternal circulation, such as hormones and metabolites, and local secretions from the endometrial glands, collectively referred to as histotroph (Spencer and Bazer, 2004). There are several lines of evidence to support the importance of the uterine environment for embryogenesis. Embryos from several mammalian species generated *in vitro* exhibit abnormal metabolic activity, disrupted transcriptomic profiles, and altered ultrastructure, which collectively impair embryonic viability compared to embryos produced *in vivo* (Hansen, 2020). Experimental ablation of uterine glands, which are the primary source of luminal fluid secretions, results in compromised embryo survival and elongation in sheep (Gray et al. 2001, 2002). Disruption of the uterine environment by flushing the contents of the lumen can reduce pregnancy rate after embryo transfer in cattle (Martins et al. 2018). Furthermore, there are inherent and repeatable differences among cows in ability to maintain embryos transferred into the uterus (Geary et al. 2016; Bennett et al. 2026). Finally, the importance of temporal changes in uterine function for establishment of pregnancy has been illustrated by experiments indicating the need for temporal synchrony between mother and embryo for successful embryo transfer in several species including the cow (Randi et al. 2016).

Despite its importance, the specific composition of uterine luminal fluid during the periconceptional period remains largely undefined. A major barrier to progress is the lack of reliable techniques capable of collecting undiluted, physiologically representative samples directly from the oviduct and uterus. Most of the existing studies characterizing the uterine or oviductal metabolome in cows (Lamy et al. 2018; Tríbulo et al. 2019; Mahé et al. 2021; Silva et al. 2023; Gonella-Diaza et al. 2024), mice (Yang et al. 2020) and humans (Tokarz et al. 2020; Wang et al. 2023) have employed sample collection techniques such as flushing the uterine/oviductal lumen with fluid. An alternative method is the use of cytobrushes (Cardoso et al. 2017) to collect cell scrapings from the uterus. Both approaches introduce an unknown degree of dilution that precludes accurate determination of the absolute metabolite concentrations. Other studies have utilized *in situ* collection of fluids from tissues collected postmortem (Elhassan et al. 2001; Li R et al. 2007) but tissue hypoxia, cellular degradation, and the leakage of intracellular contents into the lumen after death can potentially alter the metabolomic profile. Catheterization of the oviduct and uterine lumen during laparotomy is possible (Hugentobler et al. 2007; Leese et al. 2008) as well as aspiration of contents of the uterine lumen by vacuum (Velazquez et al. 2010). While these methods provide undiluted samples, they require sterile conditions, specialized equipment, and highly trained personnel that pose limitations on their broader application.

Here we report the use of a mechanical method using two related devices for the collection of neat uterine fluid from the cow that can be readily used by any personnel trained in techniques for passing pipettes or catheters across the cervix *via* transrectal manipulation. There were four objectives of the current experiments. The first was to assess the efficacy of collecting neat uterine fluid from cows. During the course of the study, the original device used for fluid collection was modified to simplify the procedure for uterine fluid collection. A second objective was to characterize the metabolomic profile of neat uterine luminal fluid and compare it with blood plasma. A third objective was to test whether oral administration of rumen-protected methionine alters the metabolomic profiles of neat uterine fluid in non-lactating beef cows. Finally, it was determined whether the uterine metabolome differs between two stages of the estrous cycle – estrus (day 0 relative to anticipated ovulation) and diestrus (day 7 after anticipated ovulation) in non-lactating beef cows. We hypothesized that the uterine metabolome of dairy cows would differ from plasma, oral administration of rumen-protected methionine would increase the plasma and intrauterine methionine concentration, and the metabolome of the uterine fluid from day 0 would be different from day 7. Effects of rumen-protected methionine on the uterine fluid metabolome were evaluated because it is a common nutritional supplement in cows and has been shown to enhance milk yield and protein content (Junior et al. 2021), alter the embryonic transcriptome (Peñagaricano et al. 2013) and reduce pregnancy loss in multiparous cows (Toledo et al. 2017). Samples from both experiments were analyzed using targeted mass spectrometry to quantify absolute concentrations of a broad range of metabolites to provide novel insights into the metabolic landscape of the uterine environment during critical reproductive time points.

## Materials and methods

### Animal care

All procedures involving animals were approved by the Animal Care and Use Committee of the University of Florida and were performed following the relevant guidelines and regulations.

### Experiment 1 – Uterine metabolome of postpartum dairy cows

**Figure 1.**
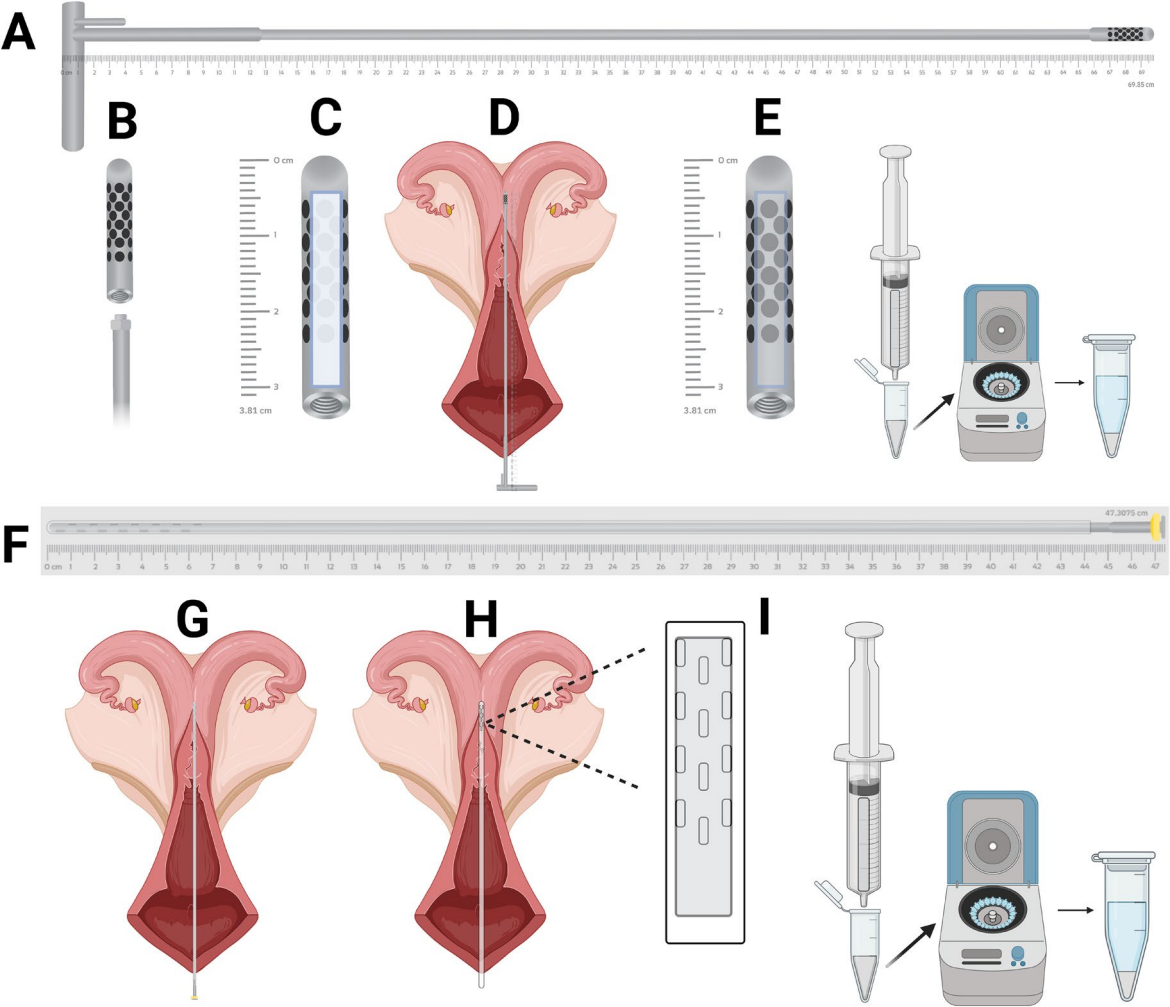
Schematic diagrams of the tools used to collect neat uterine fluid from cows in experiments 1 and 2. In experiment 1 (A–E), a custom transcervical device with a cellulose wick was inserted into the uterine body *via* the cervix. Neat uterine fluid was extracted from the wick using an insulin syringe. The supernatant was collected after centrifugation. In experiment 2 (F–I), a modified artificial insemination sheath with perforations in the tip was inserted into the uterus. A polyvinyl acetate wick was introduced through the sheath, then removed from the uterus and processed as in experiment 1.

The objective of this pilot study was to evaluate the efficacy of using a custom-made instrument for collecting uterine fluid from the cow while also comparing the metabolome of neat uterine fluid with blood plasma in lactating Holstein dairy cows. Neat uterine fluid and blood samples were simultaneously collected from six multiparous dairy cows at 50 ± 5 days postpartum. This time represents a period when uterine involution should be largely complete and preceding resumption of breeding activity after calving on many farms. Cows were selected without reference to reproductive status. Cows were fed a total mixed ration containing 64 g of metabolizable methionine using MFP® feed supplement (Novus International, Chesterfield, MO, USA) as an additional methionine source. Uterine fluid was obtained using a custom-designed transcervical sampling device (Figure 1A-E). Before use, the head of the device was removed to insert a cellulose wick (Product #30-048, DeRoyal, Tazewell, TN, USA) into its perforated chamber, reassembled, and covered with a disposable sanitary chemise (SKU 27810, WTA, College Station, TX, USA). Cows were restrained in a chute and received 5 mL of 2% (w/v) lidocaine *via* epidural injection for local anesthesia. After thoroughly cleaning the perineal area, the device was guided through the cervix and positioned in the uterine body for 3–5 min to allow full saturation of the wick. The device was then removed, and the saturated wick retrieved with forceps. Uterine fluid was extracted by placing the wick into the barrel of an insulin syringe and pressing with the plunger to collect the fluid into a sterile 2 mL centrifuge tube (Electron Microscopy Sciences, Hatfield, PA, USA). The sample was centrifuged at 400 × g for 7 min to remove debris, and the supernatant was snap-frozen in liquid nitrogen and stored at −80 °C for metabolomic analysis. Concurrently, a 10 mL blood sample was collected from the coccygeal vessels into ethylenediamine tetraacetic acid containing tubes and centrifuged at 3000×*g* for 15 min to obtain plasma, which was harvested using a Pasteur pipette, and stored at −80 °C until analysis.

### Experiment 2 – Impact of day of the estrous cycle and oral administration of rumen-protected methionine on uterine metabolome of beef cows

The objective of this experiment was to test the effects of oral administration of rumen-protected methionine on the uterine metabolome of beef females on days 0 and 7 of the estrous cycle. An additional objective was to compare uterine fluid metabolite profiles at days 0 and 7 of the estrous cycle (see statistical analysis for more details). For this purpose, reproductively healthy Brahman heifers (*n* = 9) and Angus cows (*n* = 8) were enrolled. Females of the two breeds were housed at separate facilities. All animals had free access to Bahiagrass, hay, water, and mineral supplements. Estrous cycles were synchronized using a CIDR-based protocol. On day −9, each animal received 100 µg of GnRH (Factrel, Zoetis, Parsippany, NJ, USA) intramuscularly and a CIDR insert containing 1.38 g of progesterone (Eazi-Breed CIDR, Zoetis Inc., Madison, NJ, USA). On day −2, the CIDR was removed and 25 mg of prostaglandin F2α (Lutalyse, Zoetis) was administered intramuscularly. Ovulation was induced on day 0 with a second 100-µg GnRH injection. Transrectal ultrasonography of the ovaries was performed on days 0 and 7 to confirm synchronization; only those animals that responded (Brahman *n* = 7 of 9, Angus *n* = 8 of 8) to the synchronization protocol were used.

On day 0, animals were randomly assigned to receive either 24 g of rumen-protected methionine [*n* = 9; AminoShure-XM, Balchem Inc., New Hampton, NY, USA; 68% DL-methionine (w/w)] in two gelatin capsules [Su07, Torpac, Fairfield, NJ, USA; 12 g of rumen-protected methionine (i.e. 8.16 g methionine)/capsule] or two empty capsules (control; *n* = 6). The same treatment was repeated on day 7, and animals remained in their assigned groups. For oral administration of treatments, animals were restrained in a chute and a balling gun (Torpac) with the gelatin capsule was gently inserted in the gap between the molar and incisor teeth. The capsule was dispensed on the back of the tongue, and each animal was monitored to confirm that it swallowed the capsule. On both days of the estrous cycle, blood plasma from coccygeal vessels and uterine fluid was collected 12 h after bolus administration.

For collection of uterine fluid, animals were prepared as described in experiment 1. A modified artificial insemination sheath with perforations at the tip (Figure 1F–I) was used for uterine fluid collection. The modified sheath, mounted over an artificial insemination pipette and covered with a sanitary chemise, was introduced into the uterus through the vagina and cervix, and the chemise was ruptured to expose the sheath in the uterine body. After carefully withdrawing the artificial insemination pipette, a polyvinyl acetate wick (Q604202, IVALON, New London, CT, USA) was inserted into the artificial insemination sheath and held in the uterine lumen for 3–5 min. The sheath was then removed, and the wick was extracted, inserted into a 1-mL insulin syringe, and squeezed to recover the fluid into a sterile 2-mL microcentrifuge tube. Samples were processed as described in Experiment 1. A video demonstrating the process of uterine fluid collection is freely available at Mendeley Data (https://data.mendeley.com/datasets/t6mbkrmffh/3).

Usable uterine fluid samples were collected from 5 control and 4 rumen-protected methionine supplemented cows on day 0 and from 3 control and 7 rumen-protected methionine supplemented cows on day 7. Other animals were also sampled but either no fluid was recovered (*n* = 3 at day 0 and *n* = 4 at day 7) or the fluid was too viscous to be analyzed (*n* = 3 samples from day 0 and 1 sample from day 7). One of the samples from the rumen protected methionine group at day 7 was excluded from analysis due to metabolite concentrations falling below the detectable range. A blank sample derived from a f wick soaked in purified water (W-1503, Sigma-Aldrich, St. Louis, MO, USA) was also submitted for metabolomic analysis as a control. There were no metabolites from the blank wick within the detectable range of the assay (results not shown).

### Metabolite panel

Targeted metabolomic profiling of plasma and uterine fluid samples was performed using the Biocrates MxP® Quant 500 kit (Biocrates Life Sciences AG, Innsbruck, Austria), which allows the quantification of up to 630 metabolites across a wide range of biochemical classes. This includes 107 small molecule metabolites comprising amino acids (*n* = 21), amino acid-related compounds (*n* = 29), bile acids (*n* = 14), biogenic amines (*n* = 9), carboxylic acids (*n* = 7), fatty acids (*n* = 12), hormones and related compounds (*n* = 4), indoles and derivatives (*n* = 4), nucleobases and related compounds (*n* = 2), and single representatives from vitamins and cofactors, alkaloids, amine oxides, carbohydrates and related compounds, and cresols (*n* = 1 each). The remaining 523 metabolites are lipids, spanning acylcarnitines (*n* = 40), phosphatidylcholines (*n* = 76), lysophosphatidylcholines (*n* = 14), sphingomyelins (*n* = 15), ceramides (*n* = 28), dihydroceramides (*n* = 8), hexosylceramides (*n* = 19), dihexosylceramides (*n* = 9), trihexosylceramides (*n* = 6), cholesteryl esters (*n* = 22), diglycerides (*n* = 44), and triglycerides (*n* = 242). This comprehensive coverage enabled a detailed assessment of the metabolic landscape in both compartments, offering insights into systemic and local biochemical environment of the uterus.

### Metabolome of plasma and uterine fluid samples

All reagents used were liquid chromatography-mass spectrometry (LC-MS) grade water, acetonitrile, methanol, isopropanol, and formic acid, sourced from Fisher Scientific as well as ethanol, pyridine, and phenylisothiocyanate from Sigma-Aldrich. Sample preparation followed the biocrates MxP Quant 500 kit protocol. Standards and quality controls (QCs) were thawed on ice, reconstituted in 100 µL water, and mixed on an orbital shaker at 1200 rpm for 15 min. A total of 10 µL of plasma, 20 µL of uterine fluid, and 10 µL each of calibration standards, QCs, and phosphate buffer were aliquoted into a 96-well plate, followed by nitrogen drying for 30 min. Derivatization was carried out using a phenylisothiocyanate reagent mixture, with incubation at ambient temperature for 60 min. Plates were subsequently dried under nitrogen for an additional 60 min. Metabolite extraction was performed using 5 mM ammonium acetate in methanol, with samples agitated on an orbital shaker for 30 min. The resulting extracts were collected by centrifugation at 500×*g* for 2 min. For LC analysis, extracts were diluted 1:1 with water. F or flow injection analysis (FIA), 50 µL of uterine fluid extract was mixed with 450 µL of FIA solvent, and 10 µL of plasma extract with 490 µL of solvent. Plates were sealed, shaken, and placed in the autosampler. LC-MS/MS analysis was performed using a Waters I-Class Ultra-Performance Liquid Chromatography-Mass Spectrometry system coupled with a Waters Xevo-TQ-S mass spectrometer (Waters Corporation, Milford, MA, USA), equipped with the MxP Quant 500 C18 column, guard, and precolumn mixer. The gradient used water (0.2% formic acid) and acetonitrile (0.2% formic acid) over 5.80 min at 0.8–1.0 mL/min flow. Injection volumes were 5 µL (positive mode) and 15 µL (negative mode). FIA-MS/MS was run isocratically at 0.03 mL/min using the kit solvent. Data were acquired and processed in MetIDQ software with biocrates-provided QCs used for analytical validation.

### Statistical analysis

Two analyses were performed for experiment 1. The full metabolite dataset was analyzed using the MetaboAnalyst 6.0 online platform (https://www.metaboanalyst.ca/). Data were log-transformed and auto-scaled. Orthogonal partial least squares discriminant analysis was performed to construct principal component analysis (PCA) plots and to identify metabolites that differentiated sample types, with the top 25 metabolites selected based on variable importance in projection (VIP) scores. A heatmap of the top 50 discriminatory metabolites was also generated using the t-test/ANOVA option of Metabolanalyst. Differences between plasma and uterine fluid in concentrations of individual metabolites were evaluated by MetaboAnalyst using a significance threshold of false discovery rate (FDR) of 0.1.

In addition, data on selected metabolites including amino acids, amino acid-related molecules, alkaloids, amino oxides, biogenic amines, carbohydrates, nucleobases and related molecules, and sums of acylcarnitines, ceramides, cholesteryl esters, diacylglycerols, dihydroceramides, fatty acids, glycerophospholipids, glycosylceramides, sphingolipids, and triacylglycerols, were log-transformed and analyzed by analysis of variance using the generalized linear model (GLM) procedure in SAS (version 9.4; SAS Institute Inc., Cary, NC, USA). The statistical model included the fixed effect of sample type (plasma vs. uterine fluid).

For experiment 2, full metabolite profiles were analyzed by MetaboAnalyst 6.0 to compare day 0 uterine fluid to day 7 uterine fluid. The program was used to generate PCA and VIP score plots, heatmaps, and quantitative enrichment analysis based on KEGG and small molecule pathway databases. Data preprocessing followed the same steps as in Experiment 1, including log transformation, auto scaling, and application of the *p* < 0.1 for FDR. In addition, selected metabolites in uterine fluid and plasma as defined in Experiment 1 were analyzed by analysis of variance using the GLM procedure of SAS to determine the effect of methionine supplementation on the plasma and uterine metabolome at days 0 and 7 of the estrous cycle. The statistical model included fixed effects of treatment, breed and interactions and with animal nested within breed and treatment as a random effect. The statistical model for uterine fluid samples included fixed effects of treatment, day of cycle, breed and interactions and with animal nested within breed and treatment as a random effect. Data for plasma were also analyzed separately for day 0 and day 7. For all analyses, the appropriate error term was used based on calculation of expected means squares. The Pearson correlation coefficient between plasma and uterine fluid concentrations of individual amino acids was calculated using the PROC CORR procedure in SAS.

For both experiments, data are reported for selected metabolites. Statistical significance for analysis of variance was considered at *p* < 0.05 and a tendency was considered at 0.05 ≤ *p* ≤ 0.10.

## Results

### Differences in the plasma and uterine metabolome of postpartum dairy cows

**Figure 2.**
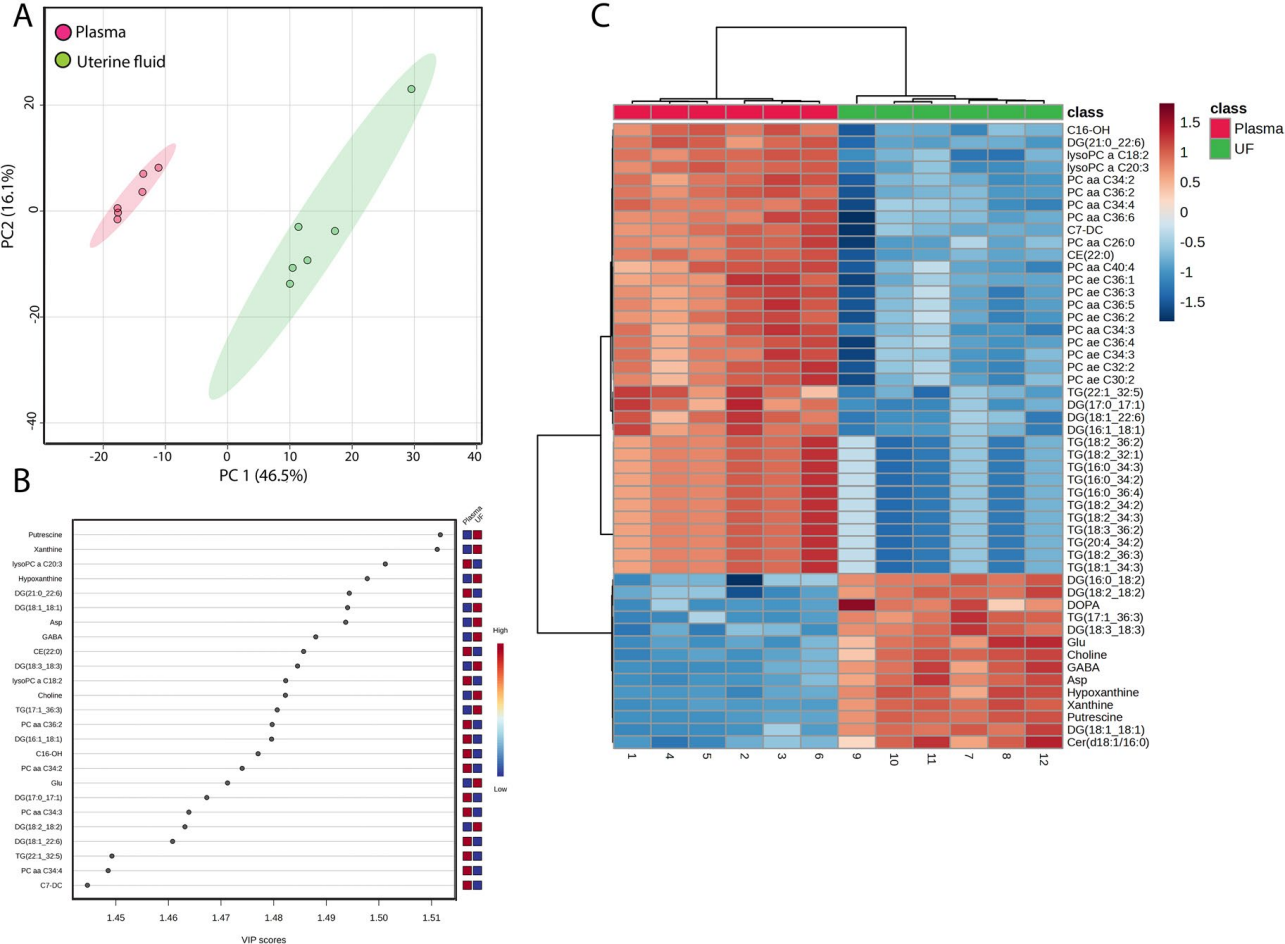
Effect of sample type (blood vs uterine fluid) on the metabolome in dairy cows at day 50 ± 5 days postpartum (*n* = 6). Shown are results of principal component (PC) analysis (A); top 25 affected metabolites by sample type based on the variable importance in projection (VIP) score (B); and heatmap of the top 50 metabolites that were discriminatory for uterine fluid vs blood (C).

Neat uterine fluid and plasma samples collected at 50 ± 5 days in milk were analyzed to determine differences in the metabolome between uterine fluid and plasma of the dairy cows. The PCA plot clearly separated uterine fluid samples from plasma samples (Figure 2A). The metabolites that contributed the most based on VIP score towards separation of uterine fluid from plasma samples were putrescine, xanthine, lysophosphatidylcholine a C20:3, and hypoxanthine (Figure 2B). A heatmap of top 50 metabolites based is presented in Figure 2C. For 36 metabolites, all of which were lipids, plasma concentrations were higher than uterine fluid concentrations. For 14 metabolites, concentrations for uterine fluid were higher than concentrations in plasma. Among these metabolites were two diacylglycerol molecules, the amino acids glutamic acid and aspartic acid, hypoxanthine, xanthine, and putrescine.

Results of analysis of variance for differences in amino acid concentrations between uterine fluid and plasma are presented in Table 1. All 21 amino acids were significantly higher for uterine fluid than plasma. Results for amino acid-related molecules are presented in Table 2. Of 29 molecules analyzed, 26 were significantly higher in uterine fluid and dihydroxyphenylalanine tended to be higher in uterine fluid (*p* = 0.083). Amino-acid-related molecules not higher in uterine fluid were nitrotyrosine and phenylalanyl-betaine.

**Table 1.** Concentration of amino acids in blood plasma and uterine fluid of dairy cows at 50 days postpartum.^a^

	Concentration, µM		
Amino acid	Plasma	Uterine fluid	*p*-Value
Alanine	249.8 ± 12.2	1549.0 ± 264.7	<0.001
Arginine	83.9 ± 4.5	286.8 ± 93.5	<0.001
Asparagine	52.2 ± 2.2	177.5 ± 19.7	<0.001
Aspartic acid	9.7 ± 0.7	2858.0 ± 202.6	<0.001
Cysteine	43.2 ± 2.1	1409.8 ± 234.8	<0.001
Cystine	31.5 ± 3.8	262.7 ± 29.9	<0.001
Glutamic acid	45.2 ± 2.3	6714.2 ± 487.4	<0.001
Glutamine	314.2 ± 17.0	1046.2 ± 253.6	<0.001
Glycine	452.0 ± 51.1	6605.7 ± 1194.1	<0.001
Histidine	68.5 ± 2.6	229.2 ± 22.5	<0.001
Isoleucine	111.4 ± 11.3	293.0 ± 33.6	<0.001
Leucine	198.7 ± 20.5	659.7 ± 64.5	<0.001
Lysine	87.8 ± 5.1	548.8 ± 50.0	<0.001
Methionine	21.4 ± 3.4	114.5 ± 10.2	<0.001
Phenylalanine	43.5 ± 2.8	144.7 ± 10.8	<0.001
Proline	112.3 ± 4.8	446.0 ± 64.5	<0.001
Serine	106.5 ± 4.6	326.0 ± 24.6	<0.001
Threonine	103.1 ± 5.3	272.2 ± 56.1	<0.001
Tryptophan	47.9 ± 2.1	110.1 ± 7.9	<0.001
Tyrosine	44.2 ± 4.1	152.2 ± 12.1	<0.001
Valine	241.2 ± 17.1	915.6 ± 181.7	<0.001

^a^ Data are raw means ± SEM. *p*-Values are from statistical analysis of log-transformed data. The number of samples is 6 each for plasma and uterine fluid.

**Table 2.** Concentration of selected amino acid-related molecules in blood plasma and uterine fluid of dairy cows at 50 days postpartum.^a^

	Concentration, µM		
Metabolite	Plasma	Uterine fluid	*p* Value
1-Methylhistidine	3.2 ± 0.3	6.0 ± 0.4	<0.001
3-Methylhistidine	3.0 ± 0.4	5.7 ± 0.7	<0.001
5-Aminovaleric acid	0.7 ± 0.07	3.8 ± 0.5	<0.001
Alpha-aminobutyric acid	8.8 ± 0.9	114.1 ± 8.7	<0.001
Alpha-aminoadipic acid	3.6 ± 0.3	18.6 ± 1.8	<0.001
Anserine	0.21 ± 0.04	0.78 ± 0.13	<0.001
Asymmetric dimethylarginine	0.6 ± 0.03	4.2 ± 0.6	<0.001
Beta-aminobutyric acid	0.09 ± 0.01	0.33 ± 0.04	<0.001
Betaine	37.5 ± 14.3	54.4 ± 26.7	0.031
Carnosine	15.3 ± 1.0	42.6 ± 7.0	<0.001
Cis-4-hydroxyproline	0.001 ± 0.001	0.04 ± 0.01	0.011
Citrulline	96.9 ± 9.3	182.2 ± 13.0	<0.001
Creatinine	56.7 ± 4.5	132.3 ± 4.9	<0.001
Dihydroxyphenylalanine	0.00046 ± 0.00046	3.75 ± 3.04	0.083
Homoarginine	2.1 ± 0.2	6.0 ± 0.7	<0.001
Homocysteine	4.3 ± 0.3	106.4 ± 20.1	<0.001
Kynurenine	4.5 ± 0.6	12.7 ± 2.8	<0.001
Methionine sulfoxide	0.6 ± 0.2	3.3 ± 0.5	<0.001
N-acetylornithine	0.004 ± 0.001	0.038 ± 0.005	<0.001
Nitrotyrosine	0.19 ± 0.02	0.24 ± 0.03	0.153
Ornithine	56.9 ± 5.4	266.5 ± 20.1	<0.001
Phenylacetylglutamine	18.1 ± 1.5	25.0 ± 2.1	0.015
Phenylalanyl-betaine	0.00145 ± 0.0002	0.00062 ± 0.0003	0.242
Proline betaine	0.2 ± 0.008	12.8 ± 3.6	<0.001
Sarcosine	4.8 ± 0.2	53.9 ± 14.8	<0.001
Symmetric dimethylarginine	0.5 ± 0.07	1.1 ± 0.1	<0.001
Trans-4-hydroxyproline	11.7 ± 2.6	44.5 ± 6.5	<0.001
Taurine	44.5 ± 6.5	853.2 ± 52.1	<0.001
Tryptophan betaine	0.0066 ± 0.001	0.074 ± 0.03	<0.001

^a^ Data are raw means ± SEM. *p*-Values are from statistical analysis of log-transformed data. The number of samples is 6 each for plasma and uterine fluid.

Attention was also paid to molecules that potentially play roles in cell signaling or have other specific biological roles (Table 3). Concentrations of β-alanine, dopamine, γ-aminobutyric acid (GABA), histamine, putrescine, serotonin, spermidine, spermine, trigonelline, trimethylamine N-oxide, hypoxanthine, xanthine, lactic acid, and choline were significantly higher in uterine fluid than plasma. Concentrations of hippuric acid, cortisol, and cortisone were not different (*p* > 0.10) between plasma and uterine fluid (Table 3).

**Table 3.** Concentration of selected biologically-active molecules in blood plasma and uterine fluid of dairy cows at 50 days postpartum.^a^

	Concentration, µM		
Metabolite	Plasma	Uterine fluid	*p*-Value
Beta-alanine^b^	2.5 ± 0.1	23.1 ± 6.3	0.001
Dopamine^b^	0.2 ± 0.01	4.3 ± 0.8	<0.001
Gamma-aminobutyric acid^b^	0.1 ± 0.01	72.8 ± 13.8	<0.001
Histamine^b^	0.1 ± 0.002	8.8 ± 2.4	<0.001
Putrescine^b^	0.06 ± 0.004	113.33 ± 13.3	<0.001
Serotonin^b^	1.4 ± 0.2	3.1 ± 0.8	0.029
Spermidine^b^	0.1 ± 0.003	1.6 ± 0.32	<0.001
Spermine^b^	0.2 ± 0.006	3.2 ± 0.5	<0.001
Trigonelline^c^	0.03 ± 0.003	4.2 ± 1.5	<0.001
Trimethylamine N-oxide^d^	6.6 ± 1.3	15.3 ± 2.9	0.030
Hypoxanthine^e^	0.04 ± 0.005	254.4 ± 55.1	<0.001
Xanthine^e^	0.06 ± 0.004	357.5 ± 68.4	<0.001
Hippuric acid^f^	78.7 ± 9.4	73.9 ± 6.0	0.783
Lactic acid^f^	857.0 ± 111.3	7661.0 ± 1311.9	<0.001
Cortisol^g^	0.03 ± 0.008	0.02 ± 0.007	0.882
Cortisone^g^	0.02 ± 0.007	0.05 ± 0.01	0.350
Choline^h^	7.6 ± 0.6	1761.7 ± 204.9	<0.001

^a^ Data are raw means ± SEM. *p*-Values are from statistical analysis of log-transformed data. The number of samples is 6 each for plasma and uterine fluid. ^b^Biogenic amines. ^c^Alkaloids. ^d^Amino oxides. ^e^Nucleobases and related. ^f^Carboxylic acid. ^g^Steroid hormone. ^h^Vitamin-like molecule.

Concentrations of specific metabolite classes were summed together. Results are shown in Table 4. Summed concentrations were significantly higher (*p* < 0.05) for uterine fluid for acylcarnitines, bile acids, carboxylic acids, ceramides, cholesterol esters, diacylglycerols, glycosylceramides and sphingolipids. There was also a tendency for concentrations of hexoses (*p* = 0.069) and summed triacylglycerols (*p* = 0.055) to be higher in uterine fluid than plasma.

**Table 4.** Concentrations of hexoses and sums of concentrations of various metabolite classes in blood plasma and uterine fluid of dairy cows at 50 days postpartum.^a^

	Concentration, µM		
Metabolite	Plasma	Uterine fluid	*p*-Value
Hexoses	2969.8 ± 28.3	3851.0 ± 502.9	0.069
Acylcarnitines^b^	5.3 ± 0.3	164.5 ± 46.5	<0.001
Bile acids^b^	22.9 ± 4.9	44.5 ± 9.1	0.048
Carboxylic acids^b^	975.9 ± 107.1	7907.9 ± 1295.6	<0.001
Ceramides^b^	2.9 ± 0.3	55.0 ± 6.5	<0.001
Cholesterol esters^b^	3424.0 ± 461.1	12777.0 ± 1268.0	<0.001
Diacylglycerols^b^	8.8 ± 0.7	45.4 ± 3.1	<0.001
Dihydroceramides^b^	51.6 ± 15.8	72.3 ± 7.6	0.177
Fatty acids^b^	3311.2 ± 488.6	3898.0 ± 705.2	0.558
Glycerophospholipids^b^	2218.3 ± 152.1	2739.1 ± 320.7	0.235
Glycosylceramides^b^	12.2 ± 0.9	50.2 ± 9.6	<0.001
Sphingolipids^b^	259.2 ± 16.5	530.0 ± 51.7	<0.001
Triacylglycerols^b^	58.8 ± 11.6	122.8 ± 33.5	0.055

^a^ Data are raw means ± SEM. *p*-Values are from statistical analysis of log-transformed data. The number of samples is 6 each for plasma and uterine fluid. ^b^Total sum of respective class of metabolites.

### Impact of rumen-protected methionine supplementation on uterine metabolome

There were two objectives of the experiment: to test whether the uterine metabolome can be altered by oral administration of rumen-protected methionine and to evaluate differences in the uterine metabolome between two days of the estrous cycle: day 0 (putative day of estrus) and day 7 (diestrus; when the embryo reaches the blastocyst stage and has entered the uterus).

**Figure 3.**
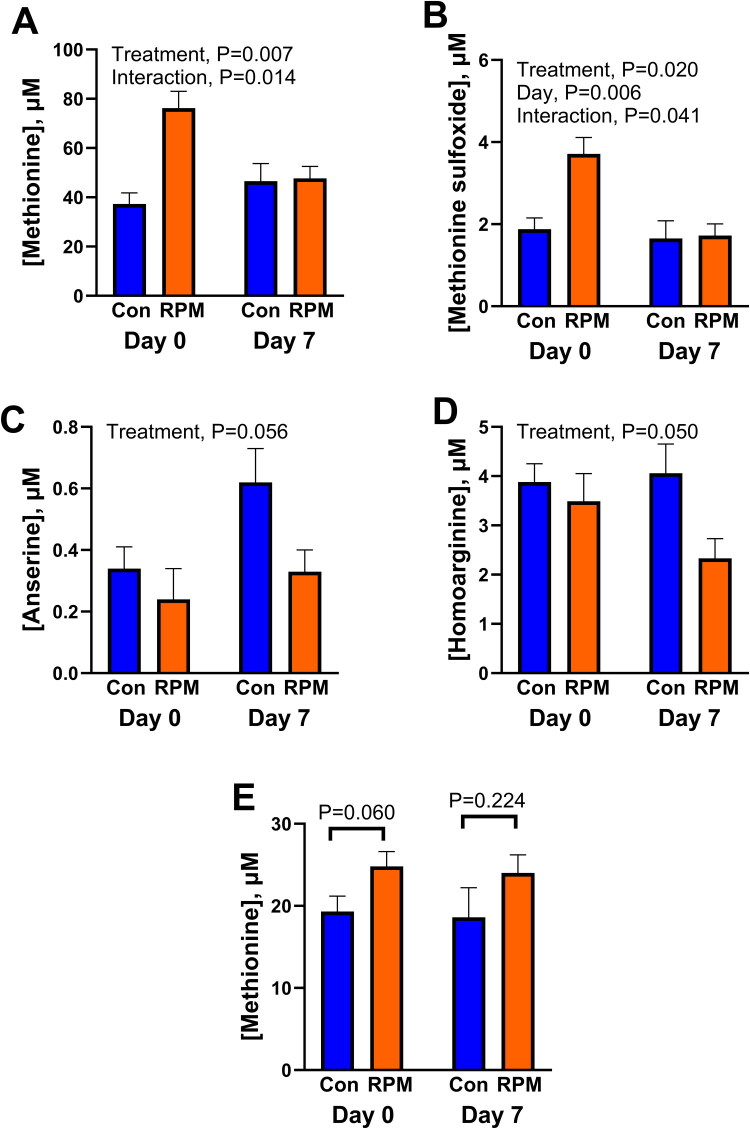
Impact of oral administration of rumen-protected methionine on the concentration of selected metabolites in uterine fluid (A–D) and plasma (E) at 12 h of bolus administration. Results shown for uterine fluid are for metabolites in which there was an effect of treatment or the treatment by day interaction. Data are presented as least-squares means ± SEM. The number of samples was 5 for control and 4 for rumen-protected methionine at day 0 and 3 for control and 6 for rumen-protected methionine at day 7.

As shown in Figure 3A, oral administration of rumen-protected methionine increased concentrations of methionine in uterine fluid 12 h after administration at day 0 but not at day 7 (day x treatment, *p* = 0.014). A similar interaction was seen for concentrations of methionine sulfoxide (Figure 3B; day x treatment, *p* = 0.0401). There were no other day by treatment interactions. Oral administration of rumen-protected methionine also tended to decrease concentrations of anserine (*p* = 0.058) and decreased concentrations of homoarginine (Figure 3D; *p* = 0.050). There were no other significant effects of rumen-protected methionine on concentrations of metabolites in uterine fluid.

Concentrations of methionine in blood at 12 h after bolus administration were also measured (Figure 3E). When data from both days were analyzed together, the effect of rumen-protected methionine (*p* = 0.160) and the interaction with day of the cycle (*p* = 0.531) were not significant. However, when data for each day were analyzed separately, rumen-protected methionine tended (*p* = 0.060) to increase concentrations of methionine at day 0 but not at day 7. At day 0, rumen-protected methionine also increased plasma concentrations of GABA (0.0134 ± 0.001 vs 0.009 ± 0.002 µM; *p* = 0.038) and choline (7.5 ± 0.7 vs 4.8 ± 0.8 µM, *p* = 0.033). At day 7, there was no significant effect of rumen-protected methionine on any metabolite.

### Effect of day of the estrous cycle on the uterine metabolome of beef cows

**Figure 4.**
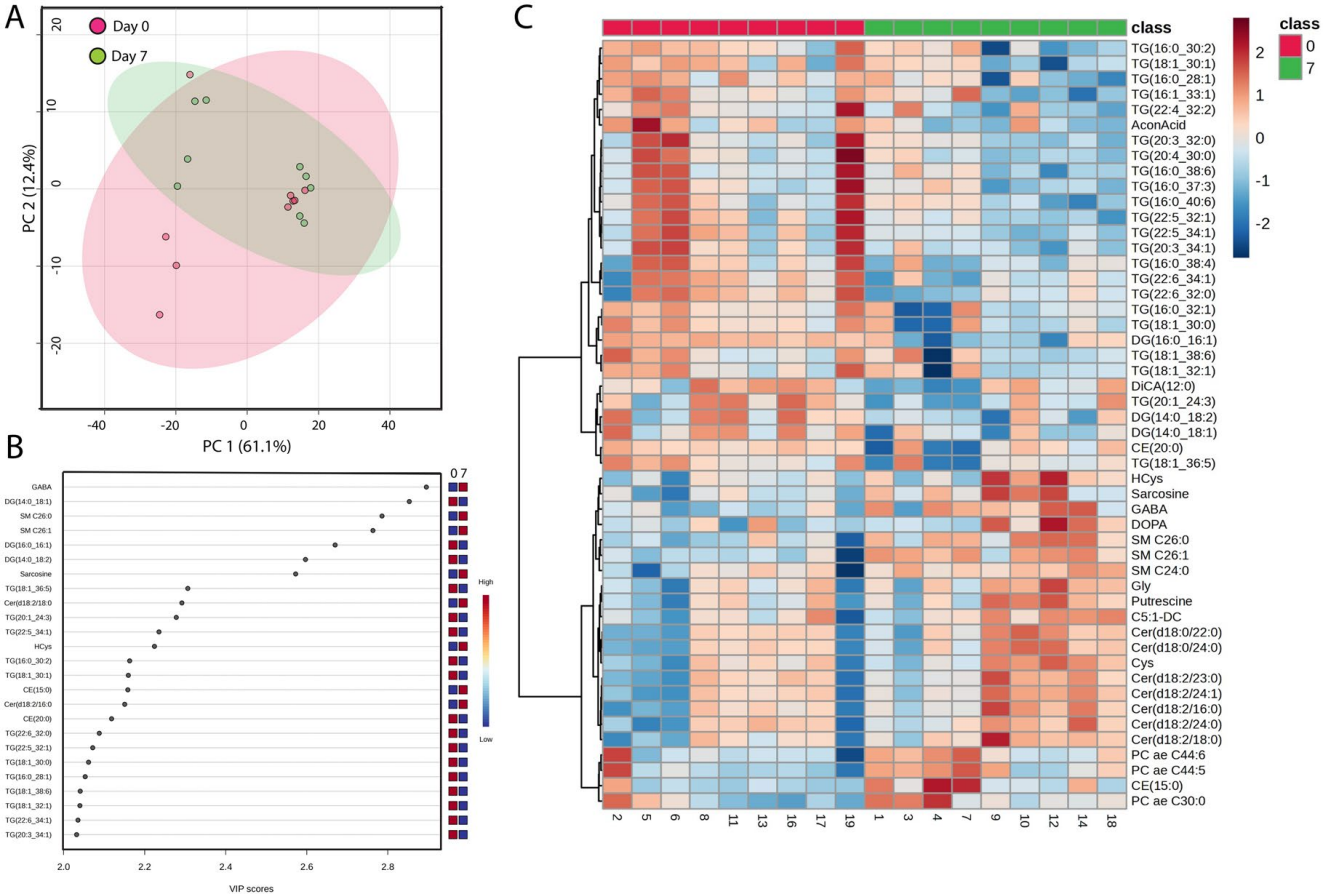
Effect of day of estrous cycle (0 vs 7) on the metabolome of uterine fluid in beef cows. Shown are results of principal component (PC) analysis (A); top 25 affected metabolites by sample type based on the variable importance in projection (VIP) score (B); and heatmap of the top 50 metabolites that discriminated day 0 from day 7 (C). The number of animals was 9 each on day 0 and day 7.

Uterine fluid samples from days 0 and 7 were incompletely separated as determined by PCA plot (Figure 4A). The most important metabolites for segregation of samples from days 0 and 7 based on VIP score are shown in Figure 4B. Lipids predominated and the four most discriminatory metabolites were GABA, diacylglycerol 14:0_18:1, sphingomyelin 26_0 and sphingomyelin C26:1.A heatmap of the top 50 metabolites based on differences from day 0 from day 7 is presented in Figure 4C. Among the metabolites that were higher at day 7 than day 0 were homocysteine, sarcosine, GABA, glycine, putrescine, cysteine, and a large number of lipids. Most of the molecules that were higher at day 0 than at day 7 were triglycerides.

**Figure 5.**
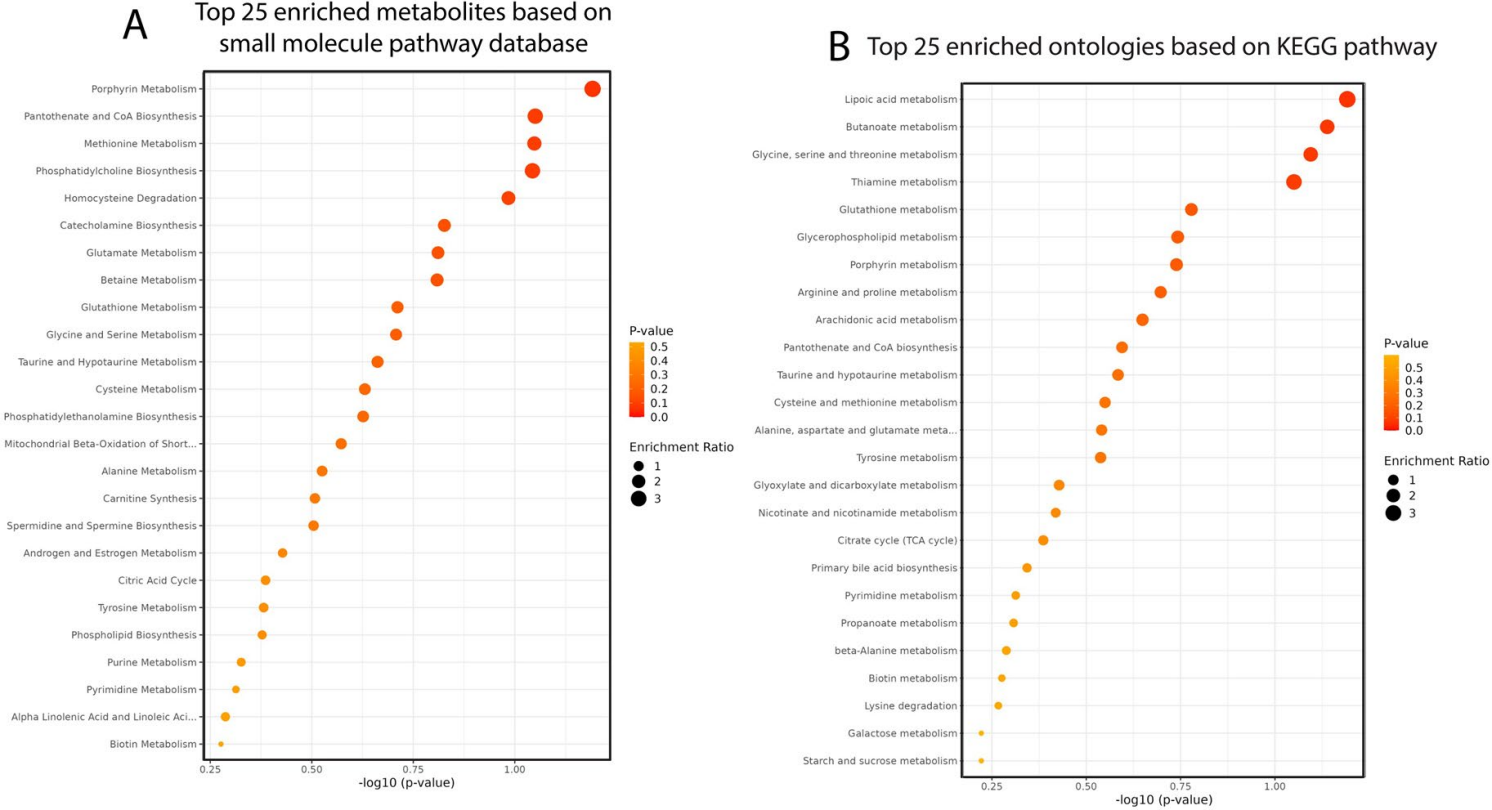
Effect of day of the estrous cycle (0 vs 7) on the metabolome of uterine fluid in beef cows on quantitative enrichment analysis of the top 25 metabolites at day 7 vs 0 based on small molecule database pathway (A) and KEGG pathway (B).

Quantitative enrichment analysis based on the KEGG and small molecule pathway databases identified several metabolic pathways that were enriched (*p* < 0.05) at day 7 as compared to day 0 (Figure 5AB). The top 5 pathways using the small molecule pathway database were porphyrin metabolism, pantothenate and CoA biosynthesis, methionine metabolism, phosphatidylcholine biosynthesis, and homocysteine degradation. The top 5 pathways using the KEGG database were lipoic acid metabolism, butanoate metabolism, glycine, serine and threonine metabolism, thiamine metabolism, and glutathione metabolism.

Effects of stage of the estrous cycle on selected metabolites were also evaluated by analysis of variance (Tables 5–8). Of 21 amino acids analyzed, two were either significantly higher (cysteine) or tended to be higher (alanine) in concentration at day 7 than day 0 and two were either significantly higher (methionine) or tended to be higher (tryptophan) in concentration at day 0 than at day 7 (Table 5). A total of 2 of 29 amino-acid related molecules were significantly affected by the day of the cycle, with asymmetric dimethylarginine being higher at day 7 than at day 0 and methionine sulfoxide being higher at day 0 (Table 6). Concentrations of selected biogenic amines and biologically-active molecules are presented in Table 7. There was no significant effect of day on any metabolite but there was a tendency for gamma-aminobutyric acid (*p* = 0.077), spermidine (*p* = 0.082) and cortisone (*p* = 0.095) to be higher at day 7 than day 0. There was also no effect of day of the estrous cycle on most summed classes of metabolites (Table 8). The exception was for ceramides and glycerophospholipids, where concentrations were higher at day 7 than day 0.

**Table 5.** Concentration of amino acids in uterine luminal fluid of beef cows at days 0 and 7 of estrous cycle.^a^

	Concentration, µM		
Amino acid	Day 0	Day 7	*p*-Value
Alanine	434.4 ± 46.2	501.2 ± 49.4	0.083
Arginine	126.9 ± 13.9	118.0 ± 14.9	0.692
Asparagine	42.4 ± 6.9	31.8 ± 7.4	0.366
Aspartic acid	726.0 ± 144.4	1077.8 ± 154.5	0.170
Cysteine	297.5 ± 221.7	833.8 ± 237.2	0.031
Cystine	67.1 ± 13.0	68.0 ± 13.9	0.966
Glutamic acid	3504.2 ± 413.8	3618.7 ± 442.7	0.863
Glutamine	441.6 ± 61.1	453.3 ± 65.3	0.905
Glycine	1478.9 ± 238.2	2208.8 ± 254.9	0.100
Histidine	79.2 ± 5.7	81.9 ± 6.1	0.765
Isoleucine	147.2 ± 11.6	114.8 ± 12.4	0.126
Leucine	211.0 ± 15.6	194.7 ± 16.7	0.521
Lysine	154.7 ± 23.0	205.7 ± 24.7	0.206
Methionine^b^	56.8 ± 2.3	47.1 ± 2.4	0.038
Phenylalanine	79.5 ± 2.3	82.9 ± 2.5	0.383
Proline	137.1 ± 14.2	151.0 ± 15.2	0.549
Serine	187.8 ± 39.1	216.3 ± 41.8	0.652
Threonine	87.9 ± 22.9	99.9 ± 24.6	0.745
Tryptophan	61.6 ± 2.4	53.0 ± 2.6	0.064
Tyrosine	78.6 ± 2.9	77.9 ± 3.1	0.873
Valine	326.5 ± 45.6	256.3 ± 48.8	0.358

^a^ Data are presented as least-squares means ± SEM. The number of samples was 9 each for days 0 and 7. ^b^The concentration of methionine in uterine fluid was affected by rumen-protected methionine treatment x day (*p* = 0.014).

**Table 6.** Concentration of selected amino acid-related molecules in uterine luminal fluid of beef cows at days 0 and 7 of estrous cycle.^a^

	Concentration, µM		
Metabolite	Day 0	Day 7	*p*-Value
1-Methylhistidine	5.9 ± 0.8	7.7 ± 0.9	0.194
3-Methylhistidine	3.0 ± 0.6	4.6 ± 0.6	0.111
5-Aminovaleric acid	1.9 ± 0.5	0.2 ± 0.6	0.135
Alpha-aminobutyric acid	31.7 ± 4.1	41.8 ± 4.4	0.169
N-Acetylornithine	0.001 ± 0.001	0.0001 ± 0.001	0.314
Alpha-aminoadipic acid	5.1 ± 0.8	5.9 ± 0.9	0.501
Anserine	0.29 ± 0.07	0.47 ± 0.08	0.153
Beta-aminobutyric acid	0.21 ± 0.02	0.19 ± 0.02	0.730
Asymmetric dimethylarginine	2.3 ± 0.1	2.9 ± 0.1	0.025
Betaine	149.7 ± 43.7	273.7 ± 46.7	0.121
Carnosine	18.6 ± 3.8	12.1 ± 4.1	0.311
Cis-4-hydroxyproline	0.02 ± 0.007	0.03 ± 0.008	0.351
Citrulline	64.6 ± 8.4	56.1 ± 9.0	0.537
Creatinine	129.9 ± 7.8	136.0 ± 8.3	0.627
Dihydroxyphenylalanine	0.08 ± 0.08	0.31 ± 0.09	0.125
Homoarginine	3.7 ± 0.3	3.2 ± 0.4	0.463
Homocysteine	38.3 ± 24.4	106.9 ± 26.1	0.124
Kynurenine	6.4 ± 2.6	6.6 ± 2.8	0.598
Methionine sulfoxide^b^	2.8 ± 0.2	1.7 ± 0.2	0.006
Nitrotyrosine	Not detected	Not detected	Not detected
Ornithine	134.7 ± 34.5	133.2 ± 36.9	0.979
Phenylacetylglutamine	16.3 ± 2.3	16.7 ± 2.5	0.920
Phenylalanyl-betaine	0.009 ± 0.001	0.010 ± 0.001	0.458
Proline betaine	4.7 ± 1.6	6.2 ± 1.7	0.433
Sarcosine	14.9 ± 17.0	63.4 ± 18.2	0.119
Symmetric dimethylarginine	0.5 ± 0.04	0.6 ± 0.04	0.374
Trans-4-hydroxyproline	32.6 ± 7.8	47.9 ± 8.4	0.258
Taurine	298.0 ± 40.0	400.7 ± 42.9	0.154
Tryptophan betaine	0.03 ± 0.01	0.04 ± 0.01	0.796

^a^ Data are least-squares means ± SEM. The number of samples was 9 each for days 0 and 7. ^b^Affected by rumen protected methionine treatment (*p* = 0.020) and the treatment by day interaction (*p* = 0.041).

**Table 7.** Concentration of selectedi biogenic amines and biologically-active molecules in uterine luminal fluid of beef cows at days 0 and 7 of estrous cycle.^a^

	Concentration, µM		
Metabolite	Day 0	Day 7	*p*-Value
Beta-alanine^b^	1.6 ± 131.6	351.4 ± 141.5	0.144
Dopamine^b^	0.4 ± 0.1	0.4 ± 0.1	0.962
Gamma-aminobutyric acid^b^	20.7 ± 5.7	39.2 ± 5.8	0.077
Histamine^b^	5.8 ± 0.4	4.8 ± 0.4	0.177
Putrescine^b^	23.6 ± 9.4	49.7 ± 10.1	0.127
Serotonin^b^	1.1 ± 0.3	0.7 ± 0.3	0.504
Spermidine^b^	1.0 ± 0.2	1.7 ± 0.2	0.082
Spermine^b^	2.5 ± 0.7	4.3 ± 0.8	0.154
Trigonelline^c^	0.05 ± 0.02	0.09 ± 0.02	0.224
Trimethylamine N-oxide^d^	38.6 ± 8.7	49.4 ± 9.3	0.453
Hypoxanthine^e^	5.1 ± 9.4	8.3 ± 10.0	0.832
Xanthine^e^	32.5 ± 23.3	23.8 ± 24.9	0.817
Hippuric acid^f^	177.4 ± 18.2	147.0 ± 19.6	0.325
Lactic acid^f^	3573.6 ± 621.5	3761.4 ± 664.9	0.851
Cortisol^g^	0.3 ± 0.1	0.5 ± 0.1	0.136
Cortisone^g^	0.3 ± 0.03	0.4 ± 0.03	0.095
Choline^h^	568.7 ± 66.7	733.7 ± 71.4	0.459

^a^ Data presented as least-squares means ± SEM. The number of samples was 9 each for days 0 and 7. ^b^Biogenic amines. ^c^Alkaloids. ^d^Amino oxides. ^e^Nucleobases and related. ^f^Carboxylic acid. ^g^Steroid hormone. ^h^Vitamin-like molecule.

**Table 8.** Concentrations of glucose and sums of concentrations of various metabolite classes in uterine luminal fluid of beef cows at days 0 and 7 of estrous cycle.^a^

	Concentration, µM		
Metabolite	Day 0	Day 7	*p*-Value
Hexoses	2977.0 ± 140.5	2577.4 ± 150.3	0.121
Acylcarnitines^b^	107.0 ± 26.7	146.7 ± 28.6	0.341
Bile acids^b^	10.3 ± 1.5	6.5 ± 1.6	0.152
Carboxylic acids^b^	3826.5 ± 648.9	3996.5 ± 694.4	0.871
Ceramides^b^	27.9 ± 6.4	57.3 ± 6.9	0.030
Cholesterol esters^b^	3456.6 ± 659.9	2388.0 ± 706.1	0.336
Diacylglycerols^b^	16.4 ± 1.2	16.2 ± 1.3	0.896
Dihydroceramides^b^	49.8 ± 10.4	46.2 ± 11.1	0.832
Fatty acids^b^	959.5 ± 1208.1	2638.7 ± 1292.6	0.403
Glycerophospholipids^b^	771.7 ± 21.1	873.3 ± 22.7	0.025
Glycosylceramides^b^	124.0 ± 5.5	123.7 ± 5.9	0.974
Sphingolipids^b^	161.0 ± 16.1	180.9 ± 17.3	0.454
Triacylglycerols^b^	163.6 ± 38.4	127.3 ± 41.1	0.562

^a^ Data are presented as least-squares means ± SEM. The number of samples was 9 each for days 0 and 7. ^b^Total sum of respective class of metabolites.

**Figure 6.**
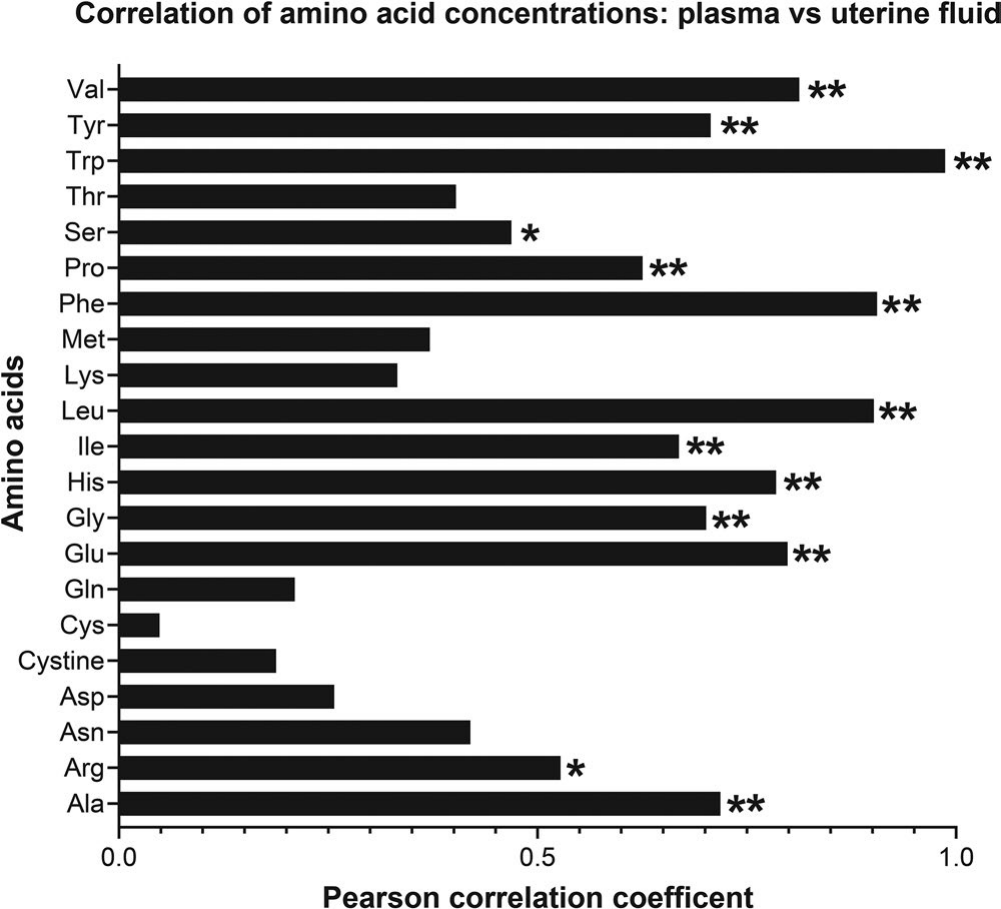
The correlation between plasma and uterine fluid concentrations of amino acids in beef cows. The number of samples was 18 for plasma and uterine fluid each. *, *p* < 0.05; **, *p* < 0.01.

Correlation analysis between plasma and uterine fluid amino acid concentrations revealed several significant positive associations (Figure 6). Strong and statistically significant correlations (*p* < 0.05) were observed for alanine (*r* = 0.73), glutamate (*r* = 0.83), glycine (*r* = 0.71), histidine (*r* = 0.78), isoleucine (*r* = 0.64), leucine (*r* = 0.88), phenylalanine (*r* = 0.90), proline (*r* = 0.63), tryptophan (*r* = 0.99), tyrosine (*r* = 0.70), and valine (*r* = 0.80). Moderate but significant correlations (*p* < 0.05) were also detected for arginine (*r* = 0.50) and serine (*r* = 0.46). In contrast, correlations for other amino acids, including asparagine, aspartate, cysteine, glutamine, lysine, methionine, threonine, and cystine, were lower and non-significant. In all cases though, correlations were positive.

## Discussion

The present study offers novel insights into the unique biochemical landscape of the uterine environment and yields several key conclusions. First, many of the detected metabolites were more concentrated in uterine fluid than in plasma, indicating specialized local regulatory mechanisms within the uterus. These may include selective transport, retention, or local synthesis of metabolites and emphasize the uterus’s active role in shaping the intrauterine milieu. Second, correlation analysis between plasma and uterine fluid amino acid concentrations revealed that several amino acids such as leucine, phenylalanine, tryptophan, valine, and glutamate were strongly correlated between compartments whereas others were weakly correlated. This observation further supports the notion that the uterine metabolome is established by contributions from the maternal systemic circulation as well as by selective local regulatory mechanisms. Third, a single oral dose of rumen-protected methionine elevated uterine methionine concentrations on day 0, demonstrating that maternal nutrition can acutely modulate the uterine environment. Finally, the uterine fluid metabolome remained relatively stable between days 0 and 7 of the estrous cycle, suggesting minimal fluctuation during the critical window of early embryo development

The presence of a large number of metabolites, including amino acids, in higher concentration in uterine luminal fluid than in plasma, in both dairy and beef cows, suggests the presence of a biologically regulated phenomenon instead of a passive diffusion of metabolites from systemic circulation. Similar observations have been made in cows (Hugentobler et al. 2007), sheep (Nancarrow, 1992), mares (Engle et al. 1984), and humans Casslén (1987), where the uterine and oviductal fluids exhibit higher concentrations of amino acids than blood.

Three major mechanisms may underlie the higher concentration of metabolites in uterine fluid: selective transport, retention, and local synthesis. The endometrium in cattle, for example, expresses transporters such as the solute carrier (SLC) family (e.g. *SLC7A1, SLC1A5*, and *SLC38A2*), glucose transporters (e.g. *GLUT1*), and ATP-binding cassette (ABC) transporters (e.g. *ABCB1, ABCG2*, and *ABCF3*) (Forde et al. 2014; Franca et al. 2017; Hoorn et al. 2024), all of which function in a regulated, directional manner to transfer nutrients. Additionally, the endometrial epithelium, which is characterized by tight junctions between epithelial cells (Murphy et al. 1982; Reardon et al. 2012; Jalali et al. 2020), can also serve as a barrier to the free movement of molecules and thus facilitate retention in the uterine lumen. In humans, for example, cultured endometrial epithelial cells formed a polarized monolayer characterized by impermeability to small molecules (Chen et al. 2016).

Another mechanism contributing to the elevated concentration of metabolites in the uterine lumen compared to peripheral blood is local synthesis within the endometrium. Bovine uterine luminal and glandular epithelial cells and stromal fibroblasts are transcriptionally active, including for genes involved in amino acid synthesis (Forde et al. 2014; Chankeaw et al. 2021). Thus, there is likely to be local production of key intermediates such as glutamine, glutamate, serine, and glycine which are important for early embryonic development (Leese et al. 2021). Similarly, genes related to lipid metabolism and de novo fatty acid synthesis and elongation (*FASN, ACACA, SCD, ELOVL5, SREBF1*), carbohydrate metabolism (*G6PD, PFKL, TKTL1*), nucleotide biosynthesis (*RRM2, DHFR*), and protein synthesis (*EIF3M, RPLP0*) are actively expressed by the bovine endometrium (Ponsuksili et al. 2012; Chankeaw et al. 2021; Alfattah et al. 2024)., The endometrium also expresses essential tricarboxylic acid cycle enzymes such as *ACO2* and *IDH1* (Bauersachs et al. 2005; Mitko et al. 2008; Forde et al. 2015) that are vital for energy production and generation of biosynthetic precursors. The bovine uterine epithelium also expresses enzymes responsible for synthesizing, catabolizing, and secreting glucose derived from glycogen stores (Sandoval et al. 2021), further enriching the metabolic environment within the uterus.

*Ex vivo* analyses demonstrate substantial metabolic transformations within uterine fluid (Simintiras et al. 2022), underscoring the presence and activity of enzymes influencing its composition. These transformations include glucose metabolism through the pentose phosphate pathway (Berg et al. 2024) and tryptophan catabolism to kynurenine (Funeshima et al. 2021). Additionally, uterine fluid contains numerous lysosomal enzymes (Hansen et al. 1985). Exosomes and extracellular vesicles are also present in uterine fluid (Kusama et al. 2021; Leal et al. 2022) and are likely to contribute to several components of the metabolome measured here such as components of lipid bilayers.

Correlation analysis between plasma and uterine fluid amino acid concentrations revealed several noteworthy associations, suggesting selective amino acid transport mechanisms or metabolic regulation within the uterine environment. Strong and statistically significant correlations were observed for alanine, glutamate, glycine, histidine, isoleucine, leucine, phenylalanine, proline, tryptophan, tyrosine, and valine, highlighting the likelihood that plasma concentrations strongly influence the availability of these amino acids in the uterine lumen. Particularly, the nearly perfect correlation for tryptophan (*r* = 0.99) and very high correlations for phenylalanine (*r* = 0.90) and leucine (*r* = 0.88) underscore their close systemic and local relationship. Conversely, concentrations of amino acids in blood such as asparagine, aspartate, cysteine, glutamine, lysine, methionine, threonine, and cystine were less strongly correlated with concentrations in uterine fluid, indicating that their concentrations within the uterine fluid might be governed to a greater extent by local synthesis, selective uptake, or utilization rather than simple equilibration with systemic plasma. In a previously published study involving beef cows, uterine concentrations of most amino acids were positively correlated with concentrations in plasma (Hugentobler et al. 2007).

Further evidence for the importance of systemic concentrations of amino acids for affecting concentrations in the uterus was obtained from evaluation of the effect of a single oral bolus of rumen-protected methionine on day 0. Such a treatment altered the uterine concentration of methionine 12 h later at day 0 of the estrous cycle but not at day 7. It is likely that a greater effect of rumen-protected methionine would have been observed if sampling was done sooner than 12 h after bolus administration. Increases in plasma methionine concentrations at 12 h after feeding were much lower than what was observed when sampling was performed 6 h after feeding (Heredia et al. 2025). In addition, the number of cows sampled was low and that may have obscured possible increase in uterine methionine concentrations after ingestion of rumen-protected methionine.

Given methionine’s central role in one-carbon metabolism and methylation *via* S-adenosylmethionine (Clare et al. 2019), changes in uterine methionine availability through feeding may have lasting consequences for embryonic epigenetic programming. Prior research supports this idea: varying concentrations of methionine in the embryo culture medium altered blastocyst DNA methylation in one study (Clare et al. 2021) and number of trophectoderm cells, neutral lipid content and apoptosis in bovine blastocysts in another (Sagheer et al. 2025). Similarly, methionine supplementation during the periconceptional period altered blastocyst gene expression (Peñagaricano et al. 2013), and increased stature of the resulting calves while also causing some changes in gene expression in muscle tissue in cows (Heredia et al. 2025).

In our study, concentrations of most metabolites remained stable between days 0 and 7 of the estrous cycle. Fahning et al. (1967) reported higher amino acid levels in pooled, undiluted uterine fluid from days 5–8 versus day 1, but without statistical analysis, interpretability is limited. Tríbulo et al. (2019) used untargeted mass spectrometry to analyze uterine flushes at days 0, 3, 5, and 7 and observed distinct temporal patterns depending on the metabolite. Differences from our findings may reflect inherent differences among animals, postmortem sampling, sample size, and the effects of uterine flushing on metabolite composition. While the overall uterine metabolome was relatively stable from day 0 to day 7, specific metabolites were enriched at day 7 including cysteine that has been reported to enhance blastocyst formation (Caamaño et al. 1998).

There was a diverse array of other bioactive metabolites in the uterine lumen, including β-alanine, dopamine, histamine, serotonin, spermidine, spermine, trigonelline, trimethylamine N-oxide, hypoxanthine, xanthine, lactic acid, hippuric acid and methionine sulfoxide. Many of these were also identified in uterine flushing from dairy cows (Tríbulo et al. 2019). These molecules could potentially regulate either development of the embryo or modify cellular function within the uterus. Genes encoding receptors for dopamine, GABA, histamine, and serotonin are expressed in bovine preimplantation embryos (Jiang et al. 2014; Sang et al. 2021). β-Alanine functions as an organic osmolyte in preimplantation mouse embryos to regulate cell volume and ionic balance (Hammer and Baltz 2023). Combined treatment with putrescine, spermidine and spermine increased development of pig parthenogenetic embryos and decreased apoptosis (Cui and Kim 2025). In contrast, a serotonin agonist was inhibitory to development of mouse preimplantation embryos (Veselá et al. 2003) and hypoxanthine inhibited development of cultured mouse embryos (Downs and Dow, 1991).

Lactic acid emerged as the most abundant uterine metabolite in the present study. It plays an important role in cells including enhancing aerobic glycolysis, participating in cell signaling, and inducing modifications of proteins through lactylation (Gurner and Gardner, 2025). It has been proposed that lactylation of H3K18 is important for embryonic genome activation (Li J et al. 2024). Two other metabolites found in uterine fluid were hippuric acid (a benzoate–glycine conjugate) and methionine sulfoxide. Concentrations of both molecules in spent embryo culture medium have been positively associated with live births in cattle (Gomez et al. 2021).

There were two major limitations in this study. The first was that attempts to recover usable uterine fluid were not always successful. All attempts to collect uterine fluid from dairy cows in experiment 1 were successful, either because of the device or the physiology of the cows. In experiment 2, however, uterine fluid was only obtained in 23 of 30 samplings (77% success). Moreover, four samples (three from day 0 and one from day 7) were too viscous for analysis. It is likely that methods can be found to reduce viscosity to allow analysis but the viscosity reduced the number of samples in the present experiment and introduced a possible bias in the day 0 vs 7 comparison. The second was the small number of animals utilized. As a result, it is likely that are differences in the uterine metabolome between day 0 and 7 not detected here because of the small sample size, especially given the large variability in concentrations of many metabolites. Examination of the data revealed that there were large differences in absolute concentrations of metabolites in uterine fluid between experiments. Thus, it is likely that many genetic, physiological or environmental factors can impact the uterine metabolome. Additional studies to identify drivers of the uterine milieu are needed.

In conclusion, this study provides compelling evidence that the uterus functions as a highly specialized and selectively regulated biochemical compartment. The finding that the majority of metabolites were more concentrated in uterine fluid than plasma, combined with the variation in correlation between amino acid concentrations between the two compartments, underscores that the uterine environment is a specialized, selectively regulated biochemical compartment. The temporal stability of the uterine metabolome between days 0 and 7 of the estrous cycle suggests a consistent biochemical support system during the early stages of embryo development. Importantly, the observed increase in uterine methionine following a single dose of rumen-protected methionine highlights the capacity of maternal nutrition to influence the uterine milieu. These results offer a comprehensive metabolomic atlas of uterine fluid, enhancing our understanding of uterine physiology and laying a foundational framework for improving reproductive outcomes through targeted nutritional interventions and optimized *in vitro* embryo culture conditions.

Probably the most valuable outcome of the research described here was the development of the methodology for sampling neat uterine fluid. Importantly, the procedure for collection was optimized after performing the preliminary study represented in Experiment 1 so that the procedure used for Experiment 2 is one that does not require specialized materials and that any technician proficient in artificial insemination should be able to perform. Further use of the methodology will allow a more comprehensive understanding of genetic and physiological determinants of the uterine metabolome in cattle.

## Data Availability

All raw data have been deposited in Mendeley Data and are freely available at https://data.mendeley.com/datasets/t6mbkrmffh/3.
